# Residential development alters behavior, movement, and energetics in an apex predator, the puma

**DOI:** 10.1371/journal.pone.0184687

**Published:** 2017-10-11

**Authors:** Yiwei Wang, Justine A. Smith, Christopher C. Wilmers

**Affiliations:** 1 San Francisco Bay Bird Observatory, 524 Valley Way, Milpitas, CA, United States of America; 2 Center for Integrated Spatial Research, Environmental Studies Department, University of California, Santa Cruz, CA, United States of America; 3 Department of Environmental Science, Policy, and Management, Mulford Hall, University of California, Berkeley, CA, United States of America; University of Southern Queensland, AUSTRALIA

## Abstract

Human development strongly influences large carnivore survival and persistence globally. Behavior changes are often the first measureable responses to human disturbances, and can have ramifications on animal populations and ecological communities. We investigated how a large carnivore responds to anthropogenic disturbances by measuring activity, movement behavior, and energetics in pumas along a housing density gradient. We used log-linear analyses to examine how habitat, time of day, and proximity to housing influenced the activity patterns of both male and female pumas in the Santa Cruz Mountains. We used spatial GPS location data in combination with Overall Dynamic Body Acceleration measurements recorded by onboard accelerometers to quantify how development density affected the average distances traveled and energy expended by pumas. Pumas responded to development differently depending on the time of day; at night, they were generally more active and moved further when they were in developed areas, but these relationships were not consistent during the day. Higher nighttime activity in developed areas increased daily caloric expenditure by 10.1% for females and 11.6% for males, resulting in increases of 3.4 and 4.0 deer prey required annually by females and males respectively. Our results support that pumas have higher energetic costs and resource requirements in human-dominated habitats due to human-induced behavioral change. Increased energetic costs for pumas are likely to have ramifications on prey species and exacerbate human-wildlife conflict, especially as exurban growth continues. Future conservation work should consider the consequences of behavioral shifts on animal energetics, individual fitness, and population viability.

## Introduction

Habitat conversion is a primary driver of species extinctions and increases exposure of wildlife to anthropogenic disturbances [1]. These disturbances influence many integral animal behaviors (e.g., foraging, mating, and movement) [2] and transform species interactions [3–4]. Conversion to low-density development at the wildland-urban interface is the fastest growing type of land use change in the continental United States [5] and is expected to continue expanding in the coming decades [6]. Although many species, including mammalian apex predators, continue to live at the wildland-urban interface [7], these regions may prove to be population sinks due to the increased risk of human-caused mortality or from the costs of adopting behavioral adaptations in response to human disturbances [8].

Behavioral changes by animals often provide the first measurable indication that individuals are responding to anthropogenic disturbance [9–11]. These behavioral responses can alter energetic budgets with important effects on individual fitness that may lead to population and community level changes. Movement behavior in particular carries rich information about where, when, and how an animal interacts with its surroundings, providing insight into the relationship between internal state and environmental factors [12]. Technological advances with GPS and accelerometer tracking devices now allow scientists to link animal movement behavior to caloric expenditure, which greatly increases our understanding of how animal energetics are impacted by human development at the landscape level. With the integration of accelerometers and traditional biologgers, we can monitor how natural and anthropogenic landscape structures change behavioral patterns and energy allocation in wild animals [13], with far ranging conservation implications for species living at the wildland-urban interface.

Large carnivores are frequently the first species to be lost from ecosystems as humans transform and develop landscapes [14]. Despite this, comparatively little is known about the behavioral and energetic responses of predators to development that could eventually lead to their local extirpation [15]. Large carnivores often respond to human disturbance and persecution through behavioral modifications much like prey species respond to predators [10]. Pumas (*Puma concolor*) have demonstrated behavioral responses to human developments by avoiding roads, moving quickly through developed areas, and changing temporal feeding patterns [8,16]. As human development continues to fragment previously intact landscapes, it becomes increasingly vital to understand how large carnivores adjust their behavior and energetic responses to anthropogenic perturbations. Only by better understanding these relationships can we implement protective policies that reduce human-wildlife conflict and promote their continued co-existence with humans [17].

Here we examined how human development alters daily behavior and energetics of pumas in the Santa Cruz Mountains of central California. We investigated the extent to which proximity to houses affected puma movements and daily activity budgets. These behavioral differences translate into differential energetic costs that progressively accumulate over time, which may have lasting repercussions on individual fitness[18]. We also investigated whether habitat type and time of day influenced how pumas responded to human development. In order to link behavior change to energetic impacts, we evaluated how human development affected the daily movement patterns and caloric expenditures of pumas using GPS tracks, which we calibrated using accelerometer data from a much finer temporal scale. Lastly, we explored the extent to which puma prey demands are altered in human-modified habitats and discuss potential consequences for recruitment of future generations.

## Methods

### Study species and area

Pumas are territorial, apex predators which live throughout diverse habitats in the Americas [19]. Individuals are primarily nocturnal and solitary, although females will typically raise and accompany cubs for up to 15–21 months after birth. In our study area in the Santa Cruz Mountains of California, pumas predominantly feed on black-tailed deer (*Odocoileus hemionus columbianus*, 90% by biomass), but occasionally on other species, including wild boars (*Sus scrofa*), raccoons (*Procyon lotor*) and house cats (*Felis catus*) [20].

Our 1,700 km^2^ study area encompasses a diverse landscape ranging from dense, urban development to large tracts of intact and relatively undisturbed native vegetation. Puma home ranges contain both protected and developed lands, with an average home range housing density of 21.7 ± 3.0 SE houses/km^2^ (range 4.6–51.5) [8]. Even pumas that regularly move through or near residential areas also use nearby protected areas, allowing for comparison of movement behavior across a disturbance gradient within individual pumas. The vegetation is primarily forested (e.g., woodlands, hardwood and conifer forests) and shrubland (e.g., scrub and chaparral) habitats. It is bisected by a large freeway and further crisscrossed by numerous other smaller roads providing access to rural houses and developments. The climate is Mediterranean, with precipitation concentrated between November and April, and elevation ranges from sea level to 1155m.

### Data collection

We captured 22 wild pumas (11 males, 11 females) from June 2010—March 2013 using trailing hounds, cage traps, or leg hold snares. Each animal was tranquilized using Telazol at a concentration of 100mg/mL (3.3–6.0 mg/kg estimated body weight) and outfitted with a GPS/VHF collar (3.7 kg; Model GPS Plus 1D, Vectronics Aerospace, Berlin, Germany). Six of the 22 animals were also equipped with a custom-built archival 3-axis accelerometer sampling continuously at 64Hz when activated [21]. The tri-axial accelerometer was mounted such that the x-axis was parallel to the anterior-posterior plane of the animal, the y-axis to the transverse plane, and the z-axis to the dorsal-ventral plane.

Accelerometers on pumas were programmed to record at a duty-cycle of 2 days on and five days off to maximize battery life. The GPS was programmed to acquire locations every 15 minutes during a 24-hour intense sampling period starting from noon one day each week. The Animal Care and Use Committee at UC Santa Cruz approved all animal-handling procedures (Protocols Wilmc0709 and Wilmc1101).

### Data processing

During each 15-minute GPS sampling interval, we assigned one behavioral state (active or inactive) to each collared individual and considered these states to be mutually exclusive. We considered any distance greater than 70m between successive 15 minute GPS fixes to be an active period, and a distance smaller than 70m to be an inactive period. We used accelerometer measurements to determine the distance cutoff between activity states as follows. We used a random forest algorithm described in Wang et al. [22] to categorize 2-second increments of accelerometer measurements into mobile or non-mobile behaviors. These were then aggregated into 15-minute observation periods to match the GPS sampling periods. After inspecting the data visually, we identified 10% activity (i.e., 10% of accelerometer measurements categorized as mobile out of 15 minutes) as the cutoff between active and inactive periods. Because of the strong linear relationship (*r* = 0.89) between accelerometer defined activity and the distance traveled between GPS fixes, 10% activity recorded by accelerometers corresponded to 70 meters between GPS fixes.

### Environmental and anthropogenic measurements

Our study animals inhabit a landscape primarily comprised of forested or shrubland habitats interspersed with developed areas. To examine how human development and habitat type affected puma behavior, we collected spatial information on buildings and habitat types surrounding each puma GPS location. Using the Geographic Information Systems program ArcGIS (v.10, ESRI, 2010), we digitized house and building locations manually from high-resolution ESRI World Imagery basemaps for rural areas and with a street address layer provided by the local counties for urban areas. For each puma GPS position recorded, we calculated the distance in meters to the nearest house. We placed circular buffers with 150m radii around each GPS location and used the California GAP analysis data [23] to categorize the local habitat as either predominantly forested or shrubland. We chose a buffer size of 150m based on a previous analysis of puma movement responses to development [24].We also classified the time each GPS location was recorded as diurnal or nocturnal based on sunset and sunrise times.

### Markov chains

We modeled puma behavior sequences as discrete-time Markov chains, which are used to describe activity states that depend on previous ones [25]. Here, we used first-order Markov chains to model a dependent relationship between the succeeding behavior and the preceding behavior. First-order Markov chains have been successfully used to describe animal behavioral states in a variety of systems, including sex differences in beaver behavior [26], behavioral responses to predators by dugongs [27], and impacts of tourism on cetacean behavior [28–29]. Because we were modeling behavior transitions with respect to spatial characteristics, we recorded the states of the puma (active or inactive) in the 15 minutes prior to and succeeding each GPS acquisition. We populated a transition matrix using these preceding and succeeding behaviors and examined whether proximity to houses influenced the transition frequencies between preceding and succeeding behavior states. Transition matrices are the probabilities that pumas remain in a behavioral state (active or inactive) or transition from one behavior state to another.

We built multi-way contingency tables to evaluate how sex (*S)*, time of day (*T*), proximity to house (*H*), and habitat type (*L*) affected the transition frequency between preceding (*B*) and succeeding behaviors (*A*). Because high-dimensional contingency tables become increasingly difficult to interpret, we first used log linear analyses to evaluate whether sex and habitat type influenced puma behavior patterns using two three-way contingency tables (Before × After × Sex, abbreviated as BAS). Log linear analyses specifically test how the response variable is influenced by independent variables (e.g., sex and habitat) by using Likelihood Ratio Tests to compare hierarchical models with and without the independent variable [25]. We found that there were strong sex differences in activity patterns because adding *S* to the model greatly increased the goodness-of-fit (*G*^*2*^) compared to the null model (Δ*G*^*2*^ = 159.8, d.f. = 1, *P*<0.0001), which assumed that succeeding behaviors only depend on preceding ones. Therefore, we evaluated data from male pumas separately from those of female pumas.

We then used another three-way contingency table for each sex to evaluate whether behavior patterns differed between habitats (*L*). We found that including habitat type significantly improved model fit for male (Δ*G*^*2*^ *=* 7.9, df = 1, *P*<0.005) but not female pumas (Δ*G*^*2*^ = 3.18, df = 1, *P* = 0.0744). Thus we evaluated three sets of data: all females, males in forests, and males in shrublands. For each dataset, we created four-way contingency tables (Before × After × House × Time) to evaluate how development and time of day affected behavioral transitions using the likelihood ratio methods described above.

Our null model (*BA*, *BHT*) is built such that succeeding behaviors (*A*) are only affected by behaviors in the previous time steps (*B*) and independent of proximity to houses and time of day. We tested whether including additional factors (proximity to house and time of day) improved model fit by comparing the null model with hierarchically more complex models. For example, the effects of proximity to housing on succeeding behaviors are evaluated by comparing the goodness-of-fit (*G*^*2*^) values for the null model and the model containing an interaction between succeeding behaviors and houses (*BAH*, *BHL*). We also tested the interaction between proximity to houses and time of day by comparing the saturated model (*BAHT*), which fits the data fully, to a less complex model without the interaction term (*BAH*, *BAT*, *BHT)*. Finally, we selected the best fitting model by minimizing the Akaike Information Crtierion (AIC) estimate.

### Behavioral budgets

We tested whether transition matrices differed when pumas were close to houses or roads using the Z test for proportions [30]. We also estimated the amount of time pumas spent in each behavioral state by conducting an eigenanalysis on the transition matrix. Because Markov chains are ergodic matrices, we used the left eigenvector of the transition matrix to estimate the proportion of time pumas spent in each state [25]. We compared these values using a Z test of proportions and calculated 95% confidence intervals using the Wilson’s score test [31].

### Puma travel and energetic costs

For each puma, we identified all 24-hour intensive sampling periods during which GPS points were recorded every 15 minutes. At a fix rate of 4 times an hour, up to 96 GPS points are recorded throughout the day, equating to a total of 95 travel segments (straight lines between consecutive points). We removed any days from analyses that were missing more than 10% (i.e., 9 points) of potential GPS fixes. We determined the linear length of all travel segments and calculated the total daily distance (*D*) in km traveled by pumas by summing all travel segments and correcting for any missing GPS fixes using the formula:

$$D_{total}=D_{summed}\times 95/n$$ (1)

in which n represents number of actual recorded segments. Next, we calculated the minimum cost of transport (*COT*, W/kg) expended daily for each puma by adapting the equation developed by Taylor et al. [32]:

$$COT=\sum_{i}^{n}10.7{\left(wt\right)}^{-0.316}\times v_{i}+6.03{\left(wt\right)}^{-0.303}$$ (2)

in which *wt* is the weight (kg) of the animal when captured and *v*_*i*_ is the velocity of travel (m/s) between consecutive GPS points. *COT* has the units Watts/kg, which we converted to kcal/kg by applying the conversion factor 4.1868 Watt = 1 cal/s.

Lastly, we estimated the minimum number of black-tailed deer, the primary prey of pumas in our area, needed to sustain each puma given their daily minimum *COT*. We calculated the daily deer biomass (*DB*) needed to fulfill each puma’s prey requirements using Eq 3 [33]:

$$DB\;\left(\frac{kg}{day}\right)=\frac{COT\;(kcal)}{1890\;(\frac{kcal}{kg})\times 0.86\times 0.88}$$ (3)

in which 1890 kcal represents the caloric content in each kg of wet deer tissue [34], and this value is then modified by multiplying it by the conversion efficiency (0.86) and the proportion of deer in a puma’s diet—here estimated as 88% [20]. Finally, we used Eq 4 to convert the daily deer biomass into an estimate of the yearly deer requirements [33]:

$$\frac{Deer}{year}=\frac{DB(\frac{kg}{day})\times 365\;days}{36.5\;kg\times 0.79}$$ (4)

in which 36.5 kg is the average weight of a black-tailed deer doe [35] and 0.79 is the edible proportion of the deer [34].

It is broadly understood that the energetic estimates generated using the equation developed by Taylor et al. [32] are the minimum estimates for *COT*. Even at 15-minute GPS sampling intervals, animals can deviate greatly from straight-line travel paths, thus expending many more kcals than estimated. In contrast, Overall Dynamic Body Acceleration (ODBA) measurements recorded by accelerometer collars, which sums the dynamic acceleration of the subject across three dimensions, provide a more precise measurement of energetic expenditure because it takes measurements at a rate of 64Hz [36]. Not all pumas were outfitted with accelerometer collars and we were unable to use ODBA alone to estimate energetic budgets. Instead we recorded ODBA values from two wild pumas whose accelerometers were active concurrent to the GPS intensive sampling periods. Using those values, we calculated the correlation between COT estimates from ODBA measurements and those estimated using velocities generated from intensive GPS sampling by Eq 2. This resulted in a correction factor that we applied to the energetic estimates of each puma in the study.

### Development influences on puma movement

To quantify puma exposure to human development, we used ArcGIS (v. 10.1, ESRI, 2012) to create buffers of 150m around all GPS points within each 24-hour intensive GPS sampling period. We then calculated the number of houses encompassed within each buffer polygon and also recorded the time of day. For each day, we recorded the average housing density individual pumas were exposed to and the average distance pumas traveled between successive GPS locations during both nocturnal and diel periods. We hypothesized that pumas would use more calories by moving faster and further through areas with more houses in order to minimize their exposure to development [24,37]. However we also predicted that this relationship might be affected by time of day because pumas may prefer to stay hidden if they are in more developed areas during the day.

We used linear mixed effects models using restricted maximum likelihood estimation with the average diurnal and nocturnal calories burned between successive GPS points as the dependent variable. To select the best model, we used a top-down model selection approach to compare models with no random terms, with random intercepts, and with both random intercepts and slopes [38]. We started by fitting a linear model that included the full complement of fixed effects terms: sex of the puma (male coded as 1 and female as 0), time of day (day coded as 1 and night as 0), the average number of houses (log-transformed to account for all distributions being bound at zero), the interactions between sex and time of day, and the interaction between time of day and housing. In a second model, puma identity was included as a factor in the model to allow for random intercepts. For the third model, we also tested whether individual pumas responded to time of day, the log average number of houses, and their interaction differently by including random slopes for those terms. We used AIC to compare the three models to determine the optimal model structure. We examined the residuals for our final model visually to identify any obvious deviations from normality.

To quantify the difference in puma energetic expenditure between areas with low and high housing density, we calculated the average caloric expenditure by individual pumas in the top and bottom housing density quartiles of their home range for both days and nights. To maximize statistical power, only pumas with a minimum of 20 day and 20 night measurements were included in this analysis. We added day and night averages to get total daily difference in caloric expenditure. We calculated the percentage increase in calories used as the total daily difference between caloric expenditure for high and low housing density divided by the average daily caloric expenditure for the individual puma. In order to conceptualize variation of human disturbances for individual pumas, we classified average housing density in the top and bottom quartiles into the following categories described by Theobald [5]: rural (greater than 0.0 and up to 0.062 houses per hectare), exurban (greater than 0.062 and up to1.236 houses per hectare), suburban (greater than 1.236 and up to 9.884 houses/hectare), or no housing. We used the package *nlme* [39] in R (v. 3.0.2, R Core Team, 2013) for all analyses.

## Results

### Log linear analyses

We recorded 78,242 GPS locations for 22 pumas, comprised of 6,967 behavioral transitions (e.g. active to inactive) for males in shrubland habitats, 11,379 transitions for males in forested habitats, and 21,977 transitions for females in all habitats. Log linear analyses revealed that both proximity to houses and time of day influenced puma activity levels, but this effect differed by sex, and by habitat type for males. Proximity to houses and time of day had a significant positive effect on the number of behavior transitions of male pumas in forests (Table 1). However, for males in forests, support for the interaction term (proximity to houses × time of day) was ambiguous because the two models had a ΔAIC of less than 0.2, indicating that they were statistically indistinguishable [40]. AIC comparison revealed that the best models for all female pumas and males in shrublands included the proximity to houses, time of day, and an interaction between the two (Table 1). This indicates that the time of day determined how pumas altered their movement patterns near development, which we discuss next.

**Table 1:** Results of log-linear analysis for all puma behavioral transition models.

Study Group	Model^a^^b^	ΔAIC^c^	Components added^a^	ΔG^2^, *df*, *P*-value
Males, Forests	Null (BA, BHT)	72.0		84, 6, —
	Previous Location × House (BAH, BHT)	63.8	BAH	71.8, 4, 0.002
	Previous location × Time (BAT, BHT)	2.2	BAT	10.2, 4, <0.001
	Previous location × Time + Previous location × House (BAT, BAH, BHT)	0.01	BAT	4.01, 2, 0.001
			BAH	4.01, 2, 0.045
	Time × House (BAHT)	0.00	TH	0, 0, 0.135
Males, Shrubland	Null (BA, BHT)	53.4		65.4, 6, —
	Previous Location × House (BAH, BHT)	54.0	BAH	62, 4, 0.002
	Previous location × Time (BAT, BHT)	8.4	BAT	16.4, 4, <0.001
	Previous location × Time + Previous location × House (BAT, BAH, BHT)	11.5	BAT	15.5, 2, <0.001
			BAH	15.5, 2, 0.64
	Time × House (BAHT)	0.00	TH	0, 0, < 0.001
Females, All habitat	Null (BA, BHT)	90.8		102.8, 6, —
	Previous Location × House (BAH, BHT)	66.6	BAH	76.4, 4, <0.001
	Previous Location × Time (BAT, BHT)	41.3	BAT	49.3, 4, <0.001
	Previous Location × Time + Previous location × House (BAT, BAH, BHT)	24.5	BAT	28.5, 2, <0.001
			BAH	28.5, 2, <0.001
	Time × House (BAHT)	0.00	TH	0, 0, < 0.001

^a^ A: Succeeding behavior; B: Previous behavior; T: Time; and H: Number of Houses.

^b^ In null models, effects of time and number of houses were assumed to be independent of behavioral transitions. Succeeding behaviors (A) are only dependent upon preceding behaviors (B), and not on time of day (T) or proximity to housing (H). Subsequent models which incorporate the housing and time covariates and their interactions are listed below the null.

^c^ ΔAIC values are in comparison to the top model for each study group.

### Behavioral budgets

**Fig 1:**
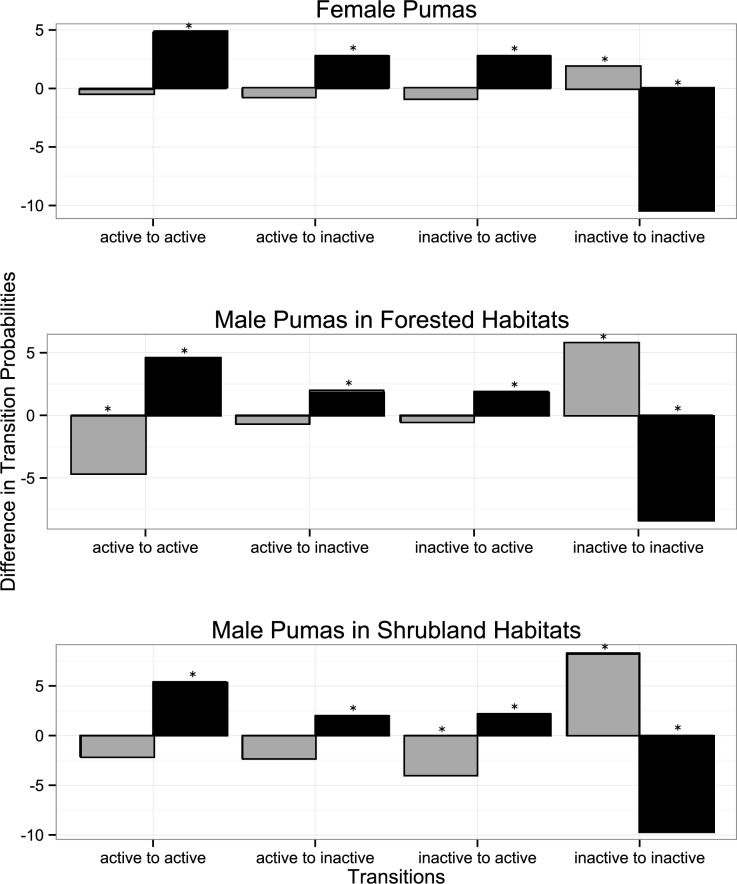
The effect of proximity to houses on the daytime (gray) and nighttime (black) transition probabilities between activity states for female pumas, male pumas in forested areas, and male pumas in shrubland habitats. Difference in transition probabilities is calculated as probability of transitioning between states when pumas are ≤150m from buildings subtracted by the probabillity of transitioning between states when pumas are >150m from buildings. A positive value means pumas are more likely to engage in those transitions when close to buildings than when further away. Asterisks above columns represent significant differences between transition probabilities close and far from houses (*P* < 0.05).

All puma behavioral transitions showed contrasting responses to housing depending on the time of day (Fig 1). At night, all pumas regardless of sex or habitat were less likely to remain inactive, more likely to remain active, and more likely to transition between behavioral states near houses. In contrast, male and female pumas were more likely to stay inactive near houses during the daytime. However, male pumas in forests were also less likely to remain active near houses in the forest during the day whereas male pumas in shrublands were unaffected.

**Fig 2:**
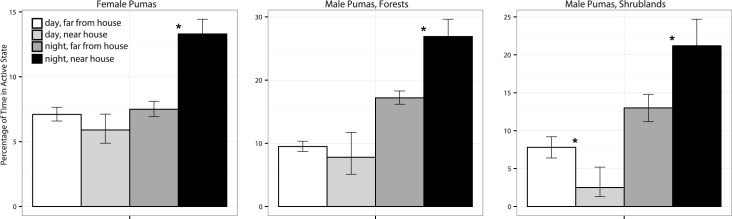
Proportion of time spent active for female pumas,male pumas in forests, and male pumas in shrublands ≤150m from buildings during the day (light gray) or night (black bars) and >150m from buildings during the day (white) or night (dark grey bars). Asterisks between paired columns represent significant differences between activity levels near houses and far from houses (*P* < 0.05). Error bars represent 95% confidence intervals.

Both male and female pumas were generally more active at night than during the day. Male pumas near houses at night were active 26.9% and 21.1% of the time in forested and shrubland habitats, respectively, compared with 17.2% and 13.2% when they weren’t close to human structures (Fig 2). Females were active 13.3% of the time when near houses at night, compared with only 7.5% when further away (Fig 2). In the daytime, puma activity was generally low, with females and males in forests exhibiting no difference in activity level in relation to proximity to houses (Fig 2). However, males in shrubland habitats were less likely to be active near houses (2.8%) than when far from houses (8%) during the day.

### Energetic costs

Our COT estimates based on ODBA measurements from accelerometers for pumas 16M and 28F showed that our energetic expenditure estimates from GPS movement data greatly underestimated caloric intake. Applying the COT formula from Taylor et al. [32] to the intensive GPS sampling period, we estimated that 16M expended 2,492 and 2,296 kcals over two days and that 28F expended 1,793 kcals. In contrast, our COT estimates from ODBA for the same three days were about 2–2.5 times higher at 6,079 and 5,492 kcals, and 3,608 kcal, respectively. We used the results from a linear regression between the COT values calculated using 15 min GPS and ODBA measurements (intercept = 8.21, slope = 1.88; *r =* 0.75) to apply corrections factor to all puma energetic calculations.

We used 19 pumas (10 males and 9 females) to evaluate movement activities and energetics over 369 24-hour intense sampling periods (216 for females and 153 for males) (Table 2). Male pumas, averaging 53.3 kg ± 7.82kg (SD), traveled a mean of 7.43 km ± 2.2 km daily and expended 5,145 kcal ± 542 kcal (after factoring the correction factor). Females, averaging 39.8 kg ± 2.73 kg, were more sedentary and traveled a mean of 4.12 km ± 0.5 km daily and expended 4,760 kcal ± 555 kcal. If a puma only subsisted on a diet of black-tailed deer, we calculated that a male puma would need to kill a minimum average of 45.5 doe equivalents/year and that a female puma would need to kill 42 doe equivalents/year.

**Table 2:** Mean (± standard error) of daily distanced traveled, daily caloric expenditure, and projected annual deer requirements of 9 female (F) and 10 male (M) pumas.

Puma ID	Days monitored	Daily distance (m)	Daily kcal/kg	Deer/year
7F	42	3236 ± 378	97.8 ± 0.6	36.3 ± 0.2
11F	22	3935 ± 489	104.5 ± 0.8	35.2 ± 0.3
18F	8	4001 ± 939	119.3 ± 1.7	40.0 ± 0.6
19F	35	3927 ± 495	107.9 ± 0.8	39.9 ± 0.3
23F	38	4389 ± 373	133.3 ± 0.8	48.0 ± 0.3
24F	15	3966 ± 462	145.0 ± 1.0	47.7 ± 0.3
25F	14	4493 ± 941	138.2 ± 2.0	48.1 ± 0.7
28F	24	4111 ± 606	129.9 ± 1.2	41.6 ± 0.4
29F	18	5060 ± 511	124.8 ± 1.0	42.2 ± 0.3
Female total	216	4132 ± 176	118.9 ± 1.1	41.6 ± 0.3
16M	12	10760 ± 1140	96.2 ± 1.5	50.4 ± 0.8
17M	8	4297 ± 706	95.0 ± 1.0	40.2 ± 0.4
22M	29	9830 ± 1091	91.2 ± 1.4	52.0 ± 0.8
26M	28	6743 ± 810	103.0 ±1.2	39.6 ± 0.5
27M	22	6853 ± 1000	99.5 ± 1.4	43.4 ± 0.6
31M	10	7047 ± 1298	94.8 ±1.8	46.1 ± 0.9
34M	17	6504 ± 727	90.1 ± 1.0	47.1 ±0.5
35M	19	4215 ± 484	97.3 ±0.7	38.8 ±0.3
36M	6	9192 ± 1874	96.4 ± 2.6	49.4 ± 1.3
37M	2	8877 ± 3	98.23 ± 0.1	48.4 ± 0.0
Male total	153	7334 ± 373	96.3 ± 0.6	45.0 ± 0.5

### Development influences on puma energetics

**Fig 3:**
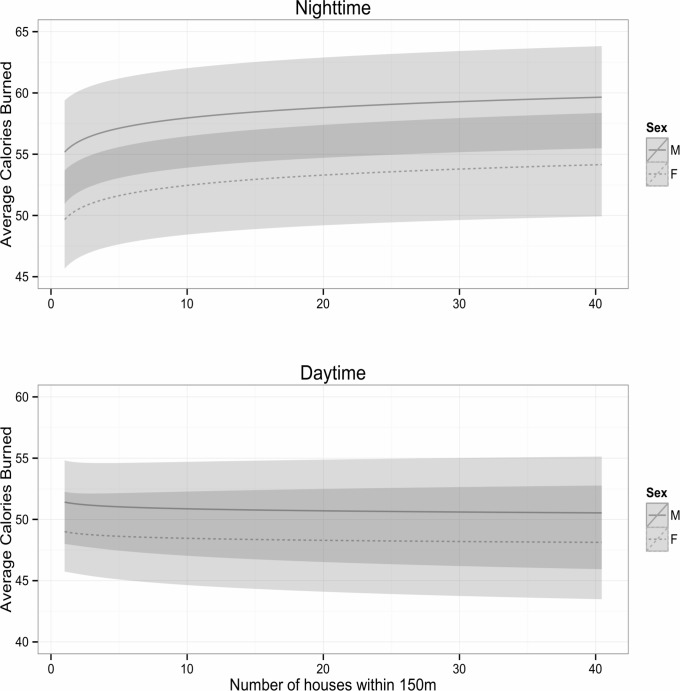
Predicted curves bounded by 95% confidence intervals relating the average calories expended between 15-minute GPS points and the average number of houses in a 150m radius around locations in nighttime and daytime. Predictions for males are indicated by the solid line and females are indicated by the dashed line.

We found that the model structure that included random intercepts and slopes for Puma ID minimized AIC values and fit the data better compared to a fixed-effects model (ΔAIC = 632) and the model with random intercepts only (ΔAIC = 23.4). The final model included all original fixed effects terms for sex, time, the log-transformed number of houses, the interaction between sex and time, and the interaction between time and number of houses (Table 3). As expected, males burned more calories than females during both nocturnal and diurnal hours (Fig 3). However, the influence of increased housing density on puma energetic expenditures differed depending on time of day, with pumas burning more calories between GPS points in more developed areas during nocturnal hours but not during diurnal hours.

**Table 3:** Results of final mixed effects model to predict puma activity.

Model Parameter	β	SE	t	*P*
Sex	5.50	2.84	1.94	0.069
Time	- 0.66	0.78	-0.85	0.395
Number of Houses (log-transformed)	1.21	0.28	4.26	< 0.001
Sex X Time	-3.10	0.97	-3.21	0.001
Time X Number of Houses	- 1.45	0.49	-2.95	0.003

Average daily caloric expenditure for individual pumas was consistently higher on days when pumas were in high housing density areas than in low housing density areas, constituting a 434.3 ± 130.3 SE kcal increase for females and a 513.3 ± 83.1 SE kcal increase for males (Table 4). These differences in average daily caloric expenditure were equivalent to a mean total percentage increase of 10.1 ± 3.1 SE% of daily kcals used by individual females and 11.6 ± 1.8 SE% of daily kcals used by males. When the increase in daily calories is converted to the extra number of deer required annually by each puma, females would need to kill an additional 3.4 deer annually to meet higher energetic requirements, and males would need to kill 4.0 more deer.

**Table 4:** Caloric difference between time spent in high and low housing density areas relative to each puma.

Puma Sex	Puma ID	Difference in kcals (day)^a^	Difference in kcals (night)^a^	Total Difference (kcal)	Increase in Daily Calories (%)^b^	Change in annual deer consumption	Bottom 25% Housing Density^c^	Top 25% Housing Density^c^
Female	23F	-754.5	809.5	55.1	1.1	0.4	Rural	Suburban
	11F	94.1	19.8	113.9	2.9	0.9	No Housing	Exurban
	28F	128.7	128.3	256.9	5.7	2.0	No Housing	Exurban
	7F	124.9	206.3	331.2	8.3	2.6	No Housing	Exurban
	19F	509.1	10.5	519.6	12.3	4.0	No Housing	Rural
	25F	54.7	736.1	790.9	16.2	6.2	No Housing	Exurban
	29F	21.2	951.3	972.6	24.4	7.6	No Housing	Exurban
Male	26M	-23.5	328.6	305.1	7.2	2.4	No Housing	Exurban
	22M	114.9	288.1	403.0	7.5	3.1	No Housing	Rural
	17M	418.8	40.2	459.0	11.8	3.6	No Housing	Exurban
	27M	63.0	408.4	471.4	10.9	3.7	No Housing	Rural
	35M	2.8	543.6	546.3	12.9	4.3	No Housing	Exurban
	16M	343.1	551.9	895.0	19.3	7.0	Rural	Exurban

^a^ Differences are calculated from average caloric expendature during days and nights spent in the top and bottom quartiles of housing density per puma.

^b^ Increase in daily calories are measured as the total increase in caloric expendature divided by individual average daily caloric expendature.

^c^ Housing density classifications are derived using categories described in Theobald (2005).

## Discussion

This study explores how housing development influences puma behavior and energetics in a fragmented landscape. Our results suggest a clear relationship between proximity to houses and puma movement activity. This effect was modulated by the time of day, whereby pumas were more likely to be active and remain active when within 150m of development at night. We also found that pumas were more likely to transition between behavioral states when close to houses. These activity shifts may reflect discomfort with being in close proximity to humans and domestic animals or reaction to other abiotic disturbances from these sources, such as light pollution or human-associated sounds [41].

As we predicted, there was a significant positive relationship between distance traveled and the number of houses surrounding each puma’s travel path. This pattern resulted in greater metabolic demand associated with higher densities of residential development. Both male and female pumas moved further and expended more calories in developed areas at night but not during the day, providing evidence that puma response to development was strongly influenced by the time of day. Although pumas only increased their movement activity near houses at night, we found that this still resulted in increased net energetic expenditure. Increases in distance traveled are unlikely to be influenced by deer availability, as occupancy of deer is ubiquitous across our study site in both developed and protected areas [20].

The increases in caloric expenditure we observed could in part explain observed increases in puma kill rate in developed areas [8]. To compensate for the higher energetic costs of living in developed areas alone, we found that pumas would need to kill on average a minimum of 3.4 and 4.0 more deer annually for female and male pumas, respectively. This estimated increase is likely conservative, as we have previously found that pumas in the most developed parts of our study area kill over 20 more deer per year than pumas in less disturbed areas [8]. Higher kill requirements based on increased movement may exacerbate other behavioral influences on energetics, including changes in feeding rates and handling time of prey [8] and altered diet composition [20].

Although pumas in our study area are not legally harvested, human-caused mortality is the leading cause of death for collared pumas. Hence, even in the absence of puma hunting, which is illegal in California, high human-induced mortality rates due to depredations give pumas strong incentive to alter their behaviors to minimize contact with people. Pumas fear humans in this human-dominated ecosystem, demonstrated by immediate responses to human stimuli [41], altered feeding behavior [8,24,41], reduced occupancy of developed areas [7], and strong avoidance of development when engaged in reproductive behaviors [24]. As large tracts of land increasingly transition from undeveloped to exurban development, non-lethal human disturbances will likely continue to alter puma behavior. As demonstrated here, changes in puma movement behavior has energetic consequences. The cumulative energetic cost of all behavior change in human-dominated systems is likely to exceed even the substantial estimated energetic requirements reported here.

Increased energetic requirements are likely to disproportionalty impact females with kittens, given their higher energetic demands [34]. Kittens older than 6 months follow their mothers to kill sites to feed [42]; if these locations are close to development, their feeding times may decline in response to disturbances [8]. Additionally, females may choose daytime resting locations further away from kill sites in developed areas, thus reducing the energetic gains kittens receive from carcasses. Although we could not track kitten survival during our study, most female pumas we tracked had kittens and lived in home ranges that encompassed developed areas. Future studies that measure kitten recruitment will shed light on the added energetic and survival costs of raising kittens in human-modified landscapes.

Our approach of using GPS and accelerometer data allowed us to obtain more accurate estamates of energetic use and requirements, which were likely underestimated in previous studies using GPS or telemetry data alone. The average activity levels of our study animals (20.8%) was relatively low compared to Beier et al.’s [37] estimates of 25% diel activity for pumas in southern California. This discrepancy may be due to methodological differences; Beier et al. [37] used the radio-telemetry to estimate the locations of animals, which is characterized by lower precision and sampling in comparison to GPS data. Pumas tracked in our study have some of the lowest travel distances (4–7 km/day) of any pumas studied, traveling less than half as far as those monitored by other studies [34,43]. However, despite their relatively short travel distances, our corrected estimates of puma energetic expenditures (average of 4,760 kcal for females and 5,145 kcal for males) was nearly twice as high as those of Laundré [34] (average of 2,420 kcal for females and 3,144 kcal for males), which suggests that previous estimates of puma energetics from GPS or radio-tracked animals have considerably underestimated true field energetics. Metabolic costs derived soley from mimimum COT equations or telemetry-only tracking studies may woefully underestimate true large predator hunting costs due to their inability to account for additional energy demand associated with topographic complexity, substrate type, intermittent locomotion, maneuvering, feeding and weather [13,44,45].

Incorporating calibrated accelerometer datasets alongside GPS locations, as demonstrated here for pumas, allows for significantly finer-scale reconstruction of behavioral and energy budgets. Our accelerometer-corrected estimates for minimum annual deer consumption (42 deer/yr for females without kittens and 45.5 deer/yr for males) are likewise much higher than those predicted by Laundré [34] (14.9 deer/yr for females and 19.4 deer/yr for males). Instead, our estimates are similar to the field-estimated kill rates of 25–84 deer/yr for pumas in our population [8].

Our study provides evidence that behavioral responses to human disturbance have energetic consequences to individuals. While previous research had focused primarily on how urbanization and development affect the persistence or declines in wildlife populations, more studies now examine the behavioral responses of these species as they adapt to increased human presence [8,46]. Understanding how animal motivations and behaviors are altered by human influences can shed light on why some species can continue to persist in human dominated landscapes while others become extirpated [47,48]. New technologies such as accelerometers can reveal much more than whether or not an animal is in an area, but elucidate how successfully the individual is able to move, feed, and reproduce [49]. Increasing awareness of the consequences of human-induced behavioral change in wildlife can contribute to more robust wildland-urban interface planning and reductions in human-wildlife conflict.

Currently, exurban or low density development is the fastest growing type of land-use change in the United States [50]. As low density development fragments previously intact landscapes, it could pose significant challenges to survival for wildlife due to cummulative effects of increased non-lethal human disturbance. By incorporating energetic measurements from accelerometers, we showed the substantial consequences of these changes in behavior on energetic costs and requirements. Changes in movement activity and behavior can provide the first indications of predator energetic responses to development. Large carnivores such as pumas occupy pivotal roles in ecosystems, and changes to their behaviors can lead to demographic effects that reverberate throughout the ecological community. In addition, as energetic needs increase with development, large carnivores may switch to domestic or synanthropic prey sources, exacerbating conflict with humans and threatening carnivore survival and population persistence. For all large carnivores, accounting for human-induced behavioral change should play a larger role in any conservation management strategy.
